# Terahertz VO_2_-Based Dynamic Coding Metasurface for Dual-Polarized, Dual-Band, and Wide-Angle RCS Reduction

**DOI:** 10.3390/nano14110914

**Published:** 2024-05-23

**Authors:** Ling Wang, Feng Gao, Shuhua Teng, Tiantian Guo, Chenggao Luo, Yang Zeng

**Affiliations:** 1 School of Electronic Information, Hunan First Normal University, Changsha 410205, China; gaofeng_nudt@hnfnu.edu.cn (F.G.); tengshuhua@nudt.edu.cn (S.T.); guotiantian18@nudt.edu.cn (T.G.); 2 Key Laboratory of Hunan Province for 3D Scene Visualization and Intelligence Education, Hunan First Normal University, Changsha 410205, China; 3 College of Electronic Science and Engineering, National University of Defense Technology, Changsha 410073, China; luochenggao@nudt.edu.cn (C.L.); zengyang@nudt.edu.cn (Y.Z.)

**Keywords:** terahertz, vanadium dioxide, coding metasurface, RCS reduction

## Abstract

With the rapid development of terahertz radar technology, the electromagnetic device for terahertz radar cross-section (RCS) reduction is worth investigating. However, the existing research concentrates on the RCS reduction metasurface with fixed performance working in the microwave band. This paper proposes a terahertz dynamic coding metasurface integrated with vanadium dioxide (VO_2_) for dual-polarized, dual-band, and wide-angle RCS reduction. The simulation result indicates that by switching the state of the VO_2_ between insulator and metal, the metasurface can realize the effective RCS reduction at 0.18 THz to 0.24 THz and 0.21 THz to 0.39 THz under the left-handed and right-handed circularly polarized incident waves. When the polar and azimuth angles of the incident wave vary from 0° to 40° and 0° to 360° respectively, this metasurface can maintain a 10 dB RCS reduction. This work has potential value in the terahertz stealth field.

## 1. Introduction

The terahertz (THz) frequency band generally means 0.1 to 10 THz. Terahertz radar has attracted considerable attention recently for high resolution, small aperture, all-day, Doppler sensitivity, and anti-interference in detection and imaging [1]. Electromagnetic (EM) stealth technology aims to make the target undetectable and protect the target. It is essential in military and civilian fields. However, the rapid development of terahertz radar technology has threatened conventional stealth devices [2]. Radar cross-section (RCS) reduction is the most significant aspect of the EM stealth device [3]. Therefore, the terahertz EM device for RCS reduction is worth investigating.

The low-profile metasurface can efficiently manipulate EM waves’ amplitude, phase, and polarization. Metasurface-based EM devices, such as the absorber, polarization converter, metalens, orbital angular momentum multiplexer, and demultiplexer, have become a research highlight [4]. RCS reduction metasurfaces have made significant progress. Most research focuses on the metasurface-based low-RCS high-gain antenna [5,6,7,8,9,10]. This research introduces complex multilayer structures to reduce RCS and enhance the gain of the meta-antenna. Some of the studies work on realizing a simple structured RCS reduction metasurface [3,11,12]. Some research further optimizes the metasurface using the particle swarm optimization (PSO) algorithm, genetic algorithm (GA), and pseudorandom algorithm [13,14,15]. In addition, the conformal RCS reduction metasurface is also investigated to improve adaptability [16,17,18]. However, the existing research concentrates on the RCS reduction metasurface with fixed performance working in the microwave band.

The dynamic metasurface (reconfigurable or tunable metasurfaces) combined with active components is superior to the fixed metasurface in flexibility, such as graphene, positive–intrinsic–negative (PIN) diode, varactor diode, and liquid crystal [19,20]. The performance of the dynamic metasurface is tunable by controlling the active component. Some achievements have been made in the dynamic RCS reduction metasurface recently. Applying PIN and varactor diodes realizes the low-RCS and high-gain antenna with reconfigurable scattering patterns [21,22]. For the vertical incident wave, using graphene-based and PIN diode-based coding metasurfaces dynamically manipulates the scattering field [23,24,25,26]. To steer the scattering field, broadband, and wide-angle metasurfaces hybridized with graphene and PIN diode are investigated [27,28]. A wideband low-scattering metasurface with an in-band tunable transparent window is also studied by loading the PIN diode [29]. Nevertheless, there is little research on the terahertz dynamic RCS reduction metasurface.

This paper offers a terahertz dynamic coding metasurface integrated with vanadium dioxide (VO_2_) for dual-polarized, dual-band, and wide-angle RCS reduction. Considering the influence of the incident wave’s polar and azimuth angles, an RCS reduction formula that covers the incident angle is derived first. Then, a coding metasurface with the whale optimization algorithm (WOA) is designed. The top metal resonator embedded with VO_2_ and a middle dielectric and bottom metal plate forms the meta-atom of the metasurface. VO_2_ undergoes the insulator-to-metal transition at around 68 °C [30]. Thus, regulating the VO_2_ state can effectively change the performance of the meta-atom. The Pancharatnam–Berry (PB) phase (geometric phase) is applied to obtain the required abrupt phase under a circularly polarized (CP) incident wave [31,32]. As the meta-atom rotation angle is limited to 0° and 90°, the metasurface is valid for both left-handed circularly polarized (LCP) and right-handed circularly polarized (RCP) incident waves. The simulation result demonstrates that the metasurface can achieve a 10 dB RCS reduction from 0.18 THz to 0.24 THz (the relative bandwidth is 28.6%) and 0.35 THz to 0.43 THz (the relative bandwidth is 20.5%) under LCP and RCP incident waves. When the polar and azimuth angles of the incident wave vary from 0° to 40° and 0° to 360°, the metasurface can generally maintain the 10 dB RCS reduction. Therefore, the terahertz VO_2_-based dynamic coding metasurface can effectively implement the dual-band, dual-polarized, and wide-angle RCS reduction. This work owns potential value in the terahertz stealth field.

## 2. Design Principle

First, consider a coding metasurface arranged in a rectangular grid on the *x*o*y* plane. There are *N_x_* and *N_y_* elements in the *x*- and *y*-directions, respectively. The distances between the elements are *D_x_* and *D_y_* in the *x*- and *y*-direction. The metasurface is excited by a plane wave with a polar angle *θ_i_* and azimuth angle *φ_i_*. According to the phased array theory [33,34], and simultaneously considering the influence of the incident wave’s angle, for the radiated field at a point in space (*θ*,*φ*), the phase difference between elements consists of three parts: the abrupt phase *ϕ*_1_ introduced by the element, the phase delay *ϕ*_2_ caused by the element’s spatial position, and the phase delay *ϕ*_3_ caused by the incident wave. For the *n_x_n_y_*th element, *ϕ*_2_ and *ϕ*_3_ can be written as:

(1) $$\phi_{2}\left(n_{x},n_{y}\right)=k_{0}\sin\theta \left(\frac{2n_{x}-1}{2}D_{x}\cos\varphi +\frac{2n_{y}-1}{2}D_{y}\sin\varphi \right)$$

(2) $$\phi_{3}\left(n_{x},n_{y}\right)=k_{0}\sin\theta_{i}\left(\frac{2n_{x}-1}{2}D_{x}\cos\varphi_{i}+\frac{2n_{y}-1}{2}D_{y}\sin\varphi_{i}\right)$$

Therefore, take the amplitude *A*(*n_x_*,*n_y_*) in each element into consideration and write the array factor *F_a_* of the metasurface as:

(3) $$F_{a}\left(\theta ,\varphi \right)=\sum_{n_{x}=0}^{N_{x}}\sum_{n_{y}=0}^{N_{y}}A\left(n_{x},n_{y}\right)\exp j\left[\phi_{1}\left(n_{x},n_{y}\right)+\phi_{2}\left(n_{x},n_{y}\right)+\phi_{3}\left(n_{x},n_{y}\right)\right]$$

The wave number *k*_0_ = 2π*C/f*_0_, *C* is the wave velocity in free space, and *f*_0_ is the frequency.

Then, to optimize the RCS reduction effect, the WOA [35] is employed. The fitness function *F_fit_* is the maximum value of *F_a_*. Express *F_fit_* as:

(4) $$F_{fit}=\max\left[F_{a}\left(\theta ,\varphi \right)\right]$$

**Figure 1:**
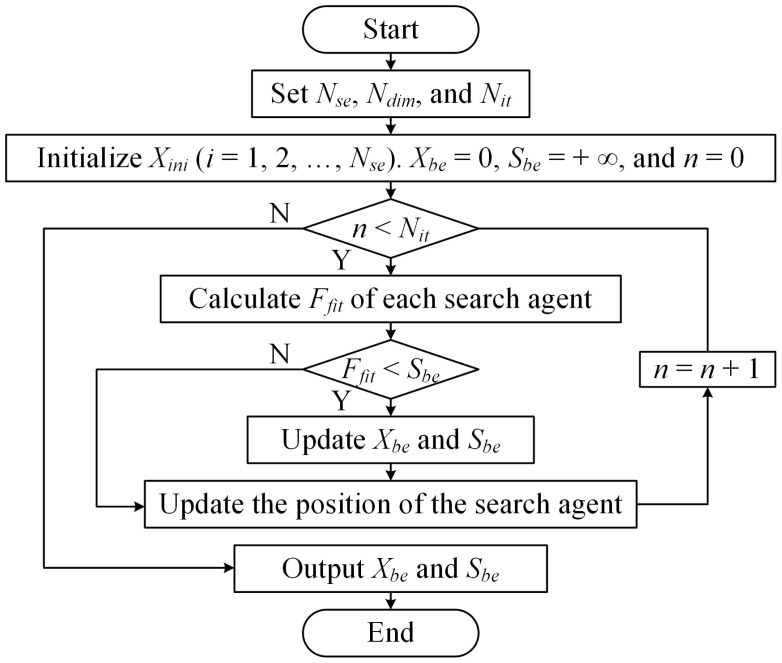
The WOA-based optimization process.

Figure 1 shows the WOA-based optimization process. Firstly, set the number of search agents *N_se_*, the number of variables *N_dim_* = *N_x_* × *N_y_*, and the maximum number of iterations *N_it_*. Then, initialize the whale population with a random coding matrix *X_ini_* (*i* = 1, 2, …, *N_se_*). *X_ini_* contains *N_dim_* elements. The elements are 0 or 1. Initialize the best search agent *X_be_* to null matrix and the best score *S_be_* to positive infinity. Next, perform iterative optimization. Calculate the *F_fit_* of each search agent using Equations (3) and (4) and update *X_be_* and *S_be_* if there is a better solution. Update the position of the search agent by encircling prey, spiral bubble-net attacking method, and search for prey. Finally, terminate the loop when the number of iterations reaches the maximum. Obtain the optimized coding matrix *X_be_* and minimum value of *S_be_*.

Furthermore, according to Equation (1), *f*_0_ is a function of *F_a_*. The coding sequence of the metasurface should change as *f*_0_ varies. Therefore, apply VO_2_ to tune the metasurface. Use the equivalent complex permittivity *ε* and conductivity *σ* to describe VO_2_ [36,37]. Based on the Drude model, write *ε* and *σ* as:

(5) $$\varepsilon \left(\omega \right)=\varepsilon_{\infty }-\frac{\omega_{p}^{2}\left(\sigma \right)}{\omega^{2}+j\gamma \omega }$$

(6) $$\sigma \left(\omega \right)=\frac{j\varepsilon_{0}\omega_{p}^{2}\left(\sigma \right)}{\omega +j\gamma }$$

(7) $$\omega_{p}^{2}\left(\sigma \right)\approx \frac{\sigma }{\sigma_{0}}\omega_{p}^{2}\left(\sigma_{0}\right)$$

*ε*_∞_ is the permittivity at high frequency, *ω_p_* (*σ*) is the plasma frequency, and the collision frequency *γ* equals 5.75 × 10^13^ s^−1^. *ω_p_* (*σ*_0_) = 1.4 × 10^15^ rad/s with *σ*_0_ = 3 × 10^3^ Ω^−1^cm^−1^ and *ε*_∞_ = 12. *σ* are taken to 200 S/m and 2 × 10^5^ S/m with the insulating and metallic VO_2_, respectively. Switching the state of the VO_2_ can theoretically realize the dynamic regulation of the metasurface.

At last, apply the PB phase principle to realize the required coding metasurface [31,32,38]. Consider an *x*-polarized or *y*-polarized wave incident to the reflective meta-atom along the negative *z*-direction. Express the incident and reflected waves as:

(8) $${\left[\begin{array}{c} E_{x}\\ E_{y} \end{array}\right]}_{\mathrm{out}}=\left[\begin{array}{cc} \cos\left(2\varphi_{rot}\right) & -\sin\left(2\varphi_{rot}\right)\\ -\sin\left(2\varphi_{rot}\right) & -\cos\left(2\varphi_{rot}\right) \end{array}\right]{\left[\begin{array}{c} E_{x}\\ E_{y} \end{array}\right]}_{\mathrm{in}}$$

*E_x_* and *E_y_* represent the linearly polarized (LP) waves. *φ_rot_* is the rotation angle of the meta-atom. The CP wave can be composed of two LP waves:

(9) $${\left[\begin{array}{c} E_{L}\\ E_{R} \end{array}\right]}_{\mathrm{in}}=\frac{1}{\sqrt{2}}\left[\begin{array}{cc} 1 & -j\\ 1 & j \end{array}\right]{\left[\begin{array}{c} E_{x}\\ E_{y} \end{array}\right]}_{\mathrm{in}}$$

(10) $${\left[\begin{array}{c} E_{L}\\ E_{R} \end{array}\right]}_{\mathrm{out}}=\frac{1}{\sqrt{2}}\left[\begin{array}{cc} 1 & j\\ 1 & -j \end{array}\right]{\left[\begin{array}{c} E_{x}\\ E_{y} \end{array}\right]}_{\mathrm{out}}$$

*E_L_* and *E_R_* are the LCP and RCP waves, respectively. Combine Equations (8)–(10):

(11) $${\left[\begin{array}{c} E_{L}\\ E_{R} \end{array}\right]}_{\mathrm{out}}=\left[\begin{array}{cc} e^{-j2\varphi_{rot}} & 0\\ 0 & e^{j2\varphi_{rot}} \end{array}\right]{\left[\begin{array}{c} E_{L}\\ E_{R} \end{array}\right]}_{\mathrm{in}}$$

Therefore, there is a −2*φ_rot_* (2*φ_rot_*) phase difference between the LCP (RCP) incident wave and the corresponding co-polarized reflected wave. The phase difference, which relates to the incident wave’s polarization state, is the PB phase. *φ_rot_* is limited to 0° and 90° to acquire the same PB phase under LCP and RCP waves.

## 3. Results and Discussion

### 3.1. Meta-Atom

**Figure 2:**
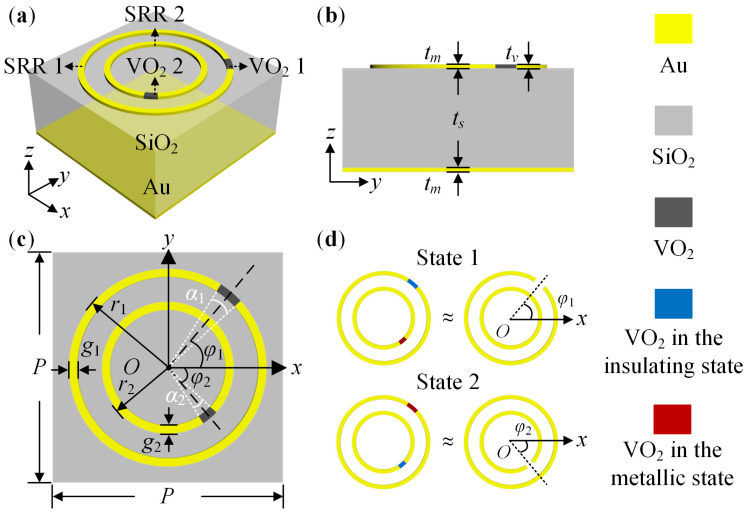
The schematic diagram of the meta-atom: (**a**) Three-dimensional structure; (**b**) Side view; (**c**) Vertical view; (**d**) The approximate equivalent structure of the Au-VO_2_ resonator.

Figure 2 displays the structure schematic diagram of the proposed metasurface’s meta-atom. Figure 2a,b indicate the metal-VO_2_ resonator, dielectric layer, and metal plate form the meta-atom. The resonator consists of two split-ring resonators (SRRs) labeled SRR 1 and SRR 2. The SRRs embed two patches of VO_2_, labeled VO_2_ 1 and VO_2_ 2. The metal is gold (Au, *σ* = 4.561 × 10^7^ S/m) with a thickness *t_m_* of 0.2 μm. The VO_2_ patch has two states: insulator (*σ* = 200 S/m) and metal (*σ* = 2 × 10^5^ S/m). The thickness *t_v_* of the VO_2_ is 0.2 μm. The dielectric is silica (SiO_2_, *ε_r_* = 3.8) with a thickness *t_s_* of 120 μm. In Figure 2c, the period *P* of the meta-atom is 280 μm. The radii *r*_1_ and *r*_2_ of SRRs are 120 μm and 80 μm, respectively. The line widths *g*_1_ and *g*_2_ are 10 μm. The angles *α*_1_ and *α*_2_ of arc-shaped VO_2_ patches are 5°. The angles between VO_2_ patches and the positive *x*-axis are *φ*_1_ and *φ*_2_, respectively. Owing to the different states of the VO_2_ patch, the Au-VO_2_ resonator can exhibit various performances. As Figure 2d suggests, when VO_2_ 1 and VO_2_ 2 are in the insulating and metallic states, respectively, the Au-VO_2_ resonator is approximately equivalent to an SRR with a rotation angle *φ*_1_ and a complete ring labeled State 1. SRR 1 works, while SRR 2 is invalid. The meta-atom rotates *φ*_1_. For State 2, the Au-VO_2_ resonator is approximately equivalent to an SRR with a rotation angle *φ*_2_ and a complete ring. SRR 1 does not work, while SRR 2 is effective. The meta-atom rotates *φ*_2_. Thus, in theory, switching the state of VO_2_ patches can tune the working frequency and the PB phase of the meta-atom under a CP incident wave.

**Figure 3:**
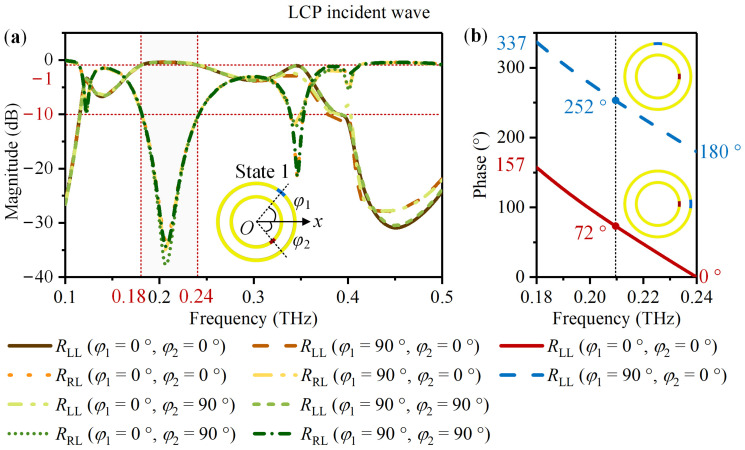
For the meta-atom in State 1 with *φ*_1_ and *φ*_2_ limited to 0° and 90°, the simulated co-polarized reflection coefficient *R*_LL_ and the cross-polarized reflection coefficient *R*_RL_ under the LCP normal incident wave: (**a**) The magnitude of *R*_LL_ and *R*_RL_; (**b**) When *φ*_1_ changes from 0° to 90° with *φ*_2_ = 0°, the phase of *R*_LL_.

**Figure 4:**
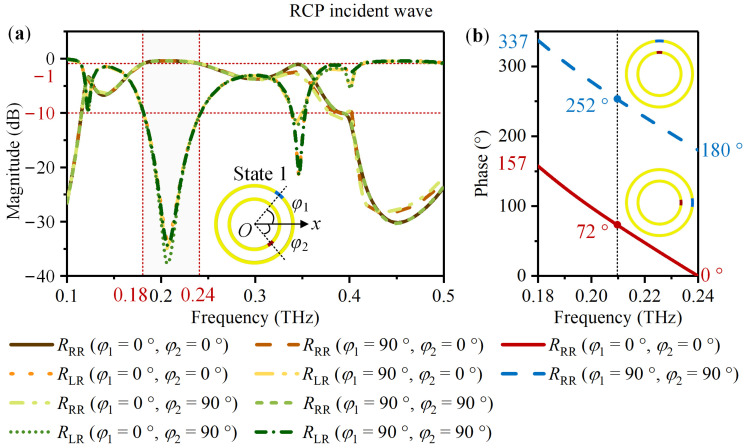
For the meta-atom in State 1 with *φ*_1_ and *φ*_2_ limited to 0° and 90°, the simulated co-polarized reflection coefficient *R*_RR_ and the cross-polarized reflection coefficient *R*_LR_ under the RCP normal incident wave: (**a**) The magnitude of *R*_RR_ and *R*_LR_; (**b**) When *φ*_1_ changes from 0° to 90° with *φ*_2_ = 0° and 90° respectively, the phase of *R*_RR_.

CST Studio Suite 2022 was used to simulate the designed meta-atom. The Frequency Domin Solver and Tetrahedral mesh type were selected. The unit cell boundary condition was applied in the *x* and *y*-directions and the open (add space) in the z-direction. The Floquet boundary was set in the *z*-direction with CP excitation. The Drude dispersion model was adapted to simulate VO_2_ with different states. *φ*_1_ and *φ*_2_ were restricted to 0° and 90° to introduce the same PB phase under LCP and RCP waves. For the meta-atom in State 1, Figure 3 and Figure 4 exhibit the simulation result. According to Figure 3a and Figure 4a, for the LCP and RCP normal incident waves, the magnitudes of the co-polarized reflection coefficients *R*_LL_ and *R*_RR_ are above −1 dB and the cross-polarized reflection coefficients *R*_RL_ and *R*_LR_ are less than −10 dB in the frequency ranging from 0.18 THz to 0.24 THz. For the LCP incident wave, take *φ*_1_ changes from 0° to 90° with *φ*_2_ = 0° as an example. Figure 3b indicates a phase difference of 180°. For the RCP incident wave, take *φ*_1_ = 0° with *φ*_2_ = 0° and *φ*_1_ = 90° with *φ*_2_ = 90° as an example. The phase difference is also about 180° based on the Figure 4b. It is thus clear that for the meta-atom in State 1, most of the CP incident wave converts into a co-polarized reflected wave within 0.18 THz to 0.24 THz. Furthermore, rotating VO_2_ 1 from 0° to 90° can introduce the same phase difference of 180° under LCP and RCP waves.

**Figure 5:**
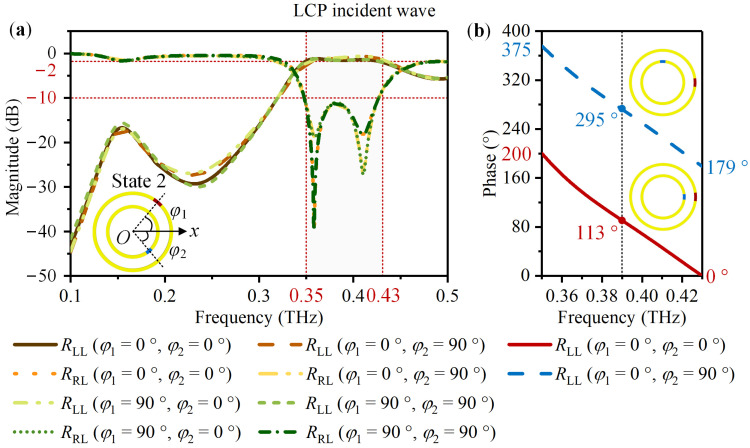
For the meta-atom in State 2 with *φ*_1_ and *φ*_2_ limited to 0° and 90°, the simulated co-polarized reflection coefficient *R*_LL_ and the cross-polarized reflection coefficient *R*_RL_ under the LCP normal incident wave: (**a**) The magnitude of *R*_LL_ and *R*_RL_; (**b**) When *φ*_2_ changes from 0° to 90° with *φ*_1_ = 0°, the phase of *R*_LL_.

**Figure 6:**
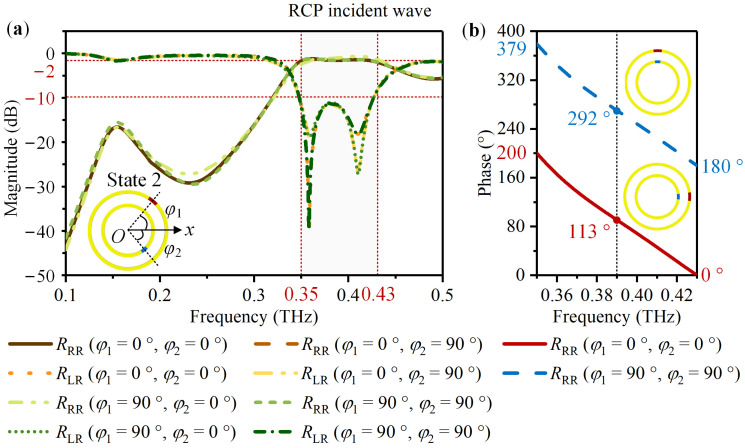
For the meta-atom in State 2 with *φ*_1_ and *φ*_2_ limited to 0° and 90°, the simulated co-polarized reflection coefficient *R*_RR_ and the cross-polarized reflection coefficient *R*_LR_ under the RCP normal incident wave: (**a**) The magnitude of *R*_RR_ and *R*_LR_; (**b**) When *φ*_2_ changes from 0° to 90° with *φ*_1_ = 0° and 90° respectively, the phase of *R*_RR_.

For State 2 with metallic VO_2_ 1 and insulating VO_2_ 2, Figure 5 and Figure 6 exhibit the simulation result. According to Figure 5a and Figure 6a, for the LCP and RCP normal incident waves, the magnitudes of *R*_LL_ and *R*_RR_ are above −2 dB, while *R*_RL_ and *R*_LR_ are less than −10 dB within 0.35 THz to 0.43 THz. For the LCP incident wave, take *φ*_2_ varies from 0° to 90° with *φ*_1_ = 0° as an example. While for the RCP incident wave, take *φ*_1_ = 0° with *φ*_2_ = 0° and *φ*_1_ = 90° with *φ*_2_ = 90° as an example. Figure 5b and Figure 6b suggest a phase difference of approximately 180°. Therefore, when the meta-atom is in State 2, most of the CP incident wave is converted into the co-polarized reflected wave in the frequency range of 0.35 THz to 0.43 THz. A same phase difference of 180° can be acquired by rotating VO_2_ 2 from 0° to 90° under LCP and RCP waves.

**Figure 7:**
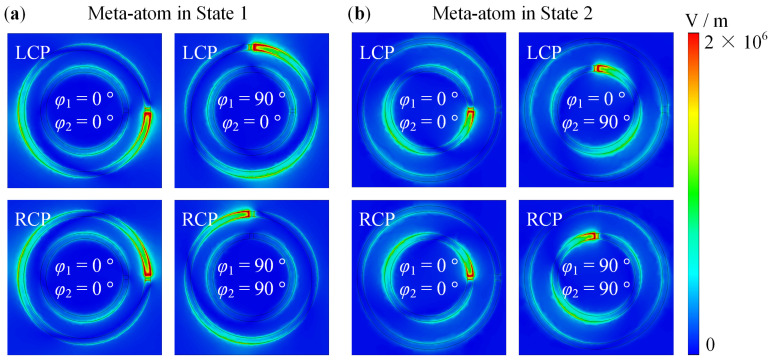
The electric field distribution of the meta-atom: (**a**) For meta-atom in State 1, the corresponding electric field distributions at 0.21 THz; (**b**) For meta-atom in State 2, the corresponding electric field distributions at 0.39 THz.

For the meta-atom in State 1 and State 2, Figure 7 shows the corresponding electric field distributions at 0.21 THz and 0.39 THz. The resonant structure switches between SRR 1 and SRR 2 by varying the states of VO_2_ 1 and VO_2_ 2. Moreover, there is a strong field at the opening of the SRR with different *φ*_1_ and *φ*_2_.

Based on the above results, adjusting the states of VO_2_ 1 and VO_2_ 2 can effectively switch the working frequency band of the meta-atom between 0.18 THz to 0.24 THz and 0.35 THz to 0.43 THz. Controlling the rotation angles *φ*_1_ and *φ*_2_ can easily change the PB phase. In addition, limiting *φ*_1_ and *φ*_2_ to 0° and 90° can bring in the same PB phase of 0° and 180° under the LCP and RCP waves.

### 3.2. Coding Metasurface

**Figure 8:**
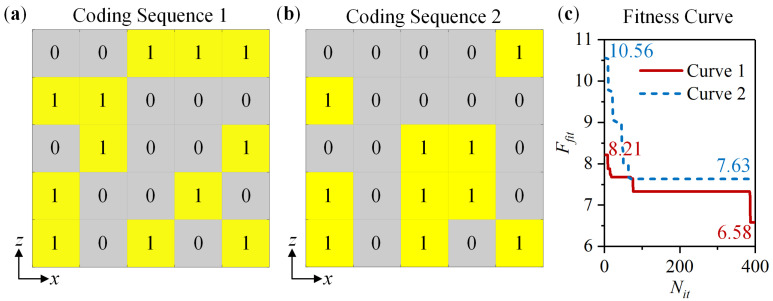
The optimal coding sequence and fitness curve: (**a**) Coding Sequence 1 at 0.21 THz; (**b**) Coding Sequence 2 at 0.39 THz; (**c**) Fitness Curve 1 at 0.21 THz and Curve 2 at 0.39 THz.

Design a 1-bit coding metasurface for verification. The metasurface consists of 5 × 5 coding elements. Each element contains 4 × 4 meta-atom. PB phases of 0° and 180° denote codes “0” and “1”, respectively. That is, the meta-atom in State 1 with *φ*_1_ = 0° and State 2 with *φ*_2_ = 0° represents “0”, while the meta-atom in State 1 with *φ*_1_ = 90° and State 2 with *φ*_2_ = 90° represents “1”. In Equations (1)–(3), *N_x_* = *N_y_* = 5 and *D_x_* = *D_y_* = 4 × *P*. Take amplitudes *A*(*n_x_*,*n_y_*) of meta-atoms equal each other. When the meta-atom operates at 0.18 THz to 0.24 THz, take *f*_0_ = 0.21 THz, *θ_i_* = 0°, and *φ_i_* = 0° as an example for optimization. While the meta-atom operates at 0.35 THz to 0.43 THz, take *f*_0_ = 0.39 THz, *θ_i_* = 0°, and *φ_i_* = 0° as an example for optimization. According to the WOA-based optimization process, set *N_se_* = 120, *N_dim_* = 25, and *N_it_* = 400. Use MATLAB R2017a software to optimize the coding sequence. The optimal Coding Sequences 1 and 2 at 0.21 THz and 0.39 THz are shown in Figure 8a and Figure 8b, respectively. Figure 8c displays the optimal fitness Curve 1 at 0.21 THz and Curve 2 at 0.39 THz. *F_fit_* decreases from 8.21 to 6.58 in Curve 1 and from 10.56 to 7.63 in Curve 2. According to Equation (3), when all of the elements are “0” or “1” in the coding sequence, *F_fit_* equals 25 theoretically. Therefore, using the optimal coding sequence can significantly reduce *F_fit_*.

**Figure 9:**
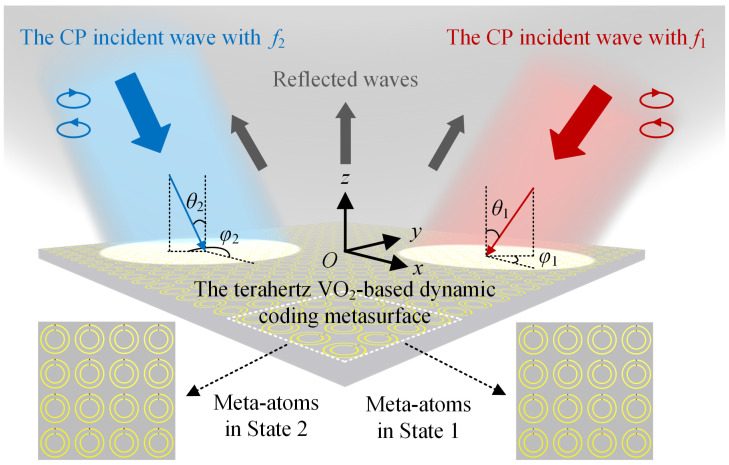
The schematic of the terahertz VO_2_-based dynamic coding metasurface.

Figure 9 shows the schematic of the terahertz VO_2_-based dynamic coding metasurface for RCS reduction. When the meta-atoms are in State 1, Coding Sequence 1 is activated, and the metasurface can realize effective RCS reduction under the LCP and RCP incident waves with the frequency *f*_1_, polar angle *θ*_1_, and azimuth angle *φ*_1_. When Coding Sequence 2 is in an active state with the meta-atoms in State 2, the metasurface can attain the RCS reduction under the CP incident wave with *f*_2_, *θ*_2_, and *φ*_2_.

**Figure 10:**
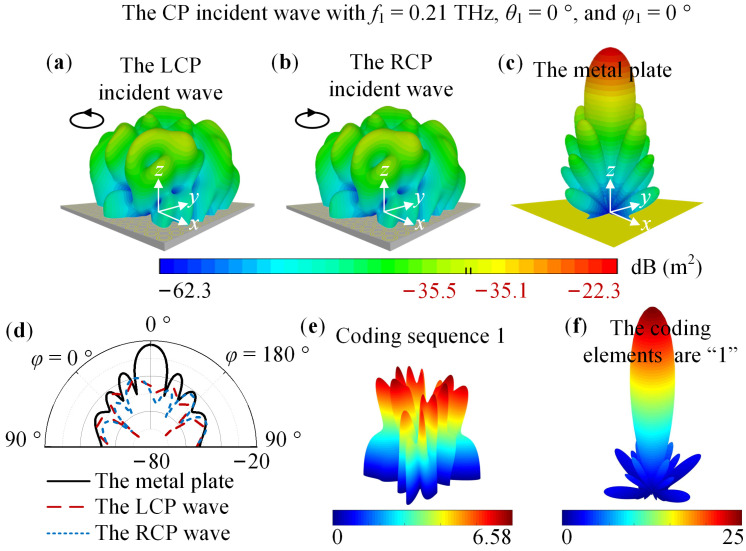
For the metasurface in State 1, the simulation result under the CP incident wave with *f*_1_ = 0.21 THz, *θ*_1_ = 0°, and *φ*_1_ = 0°. The far-field pattern simulated in CST: (**a**) The LCP incident wave; (**b**) The RCP incident wave; (**c**) The metal plate; (**d**) 2D far-field pattern; The array factor calculated in MATLAB: (**e**) Coding sequence 1; (**f**) The elements of the coding sequence are “1”.

**Figure 11:**
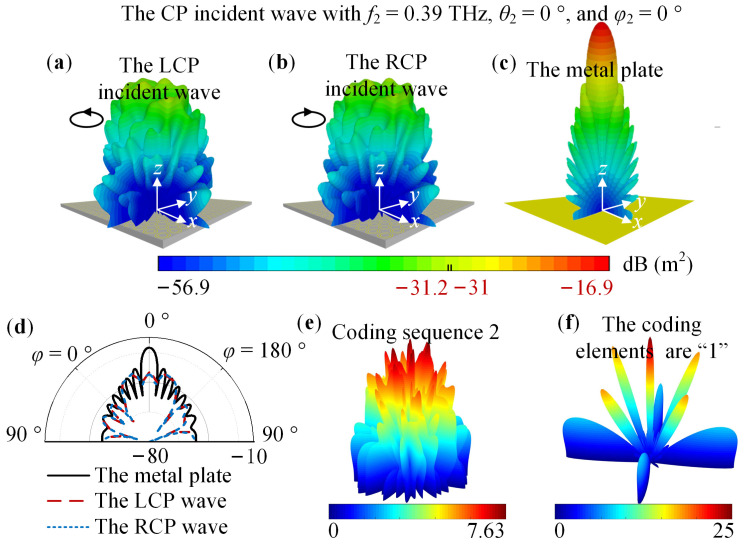
For the metasurface in State 2, the simulation result under the CP incident wave with *f*_2_ = 0.39 THz, *θ*_2_ = 0°, and *φ*_2_ = 0°. The far-field pattern simulated in CST: (**a**) The LCP incident wave; (**b**) The RCP incident wave; (**c**) The metal plate; (**d**) 2D far-field pattern; The array factor calculated in MATLAB: (**e**) Coding sequence 2; (**f**) The elements of the coding sequence are “1”.

CST Studio Suite 2022 was used to analyze the 1-bit coding metasurface. The Time Domin Solver and Hexahedral mesh type were selected. The open (add space) boundary condition was applied in the *x*, *y*, and *z*-directions, and, simultaneously, a metal plate of the same size as the metasurface was simulated for comparison. When the metasurface is in State 1, Figure 10 shows the far-field pattern under the CP incident wave with *f*_1_ = 0.21 THz, *θ*_1_ = 0°, and *φ*_1_ = 0°. For Figure 10a–c, the maximum numbers are −35.5, −35.1, and −22.3. For the metasurface in State 2, Figure 11 shows the far-field pattern under the CP incident wave with *f*_2_ = 0.39 THz, *θ*_2_ = 0°, and *φ*_2_ = 0°. For Figure 11a–c, the maximum numbers are −31, −31.2, and −16.9. In addition, Figure 10e,f and Figure 11e,f displace the corresponding array factor calculated in MATLAB. Figure 10a–d reveal that the metasurface can obtain about a 13 dB RCS reduction. Figure 11a–d exhibit an RCS reduction of 14 dB. Therefore, the simulation result demonstrates that for LCP and RCP waves with *θ_i_* = 0° and *φ_i_* = 0°, switching the state of the VO_2_ patch can simultaneously realize an RCS reduction of no less than 10 dB at 0.21 THz and 0.39 THz.

**Figure 12:**
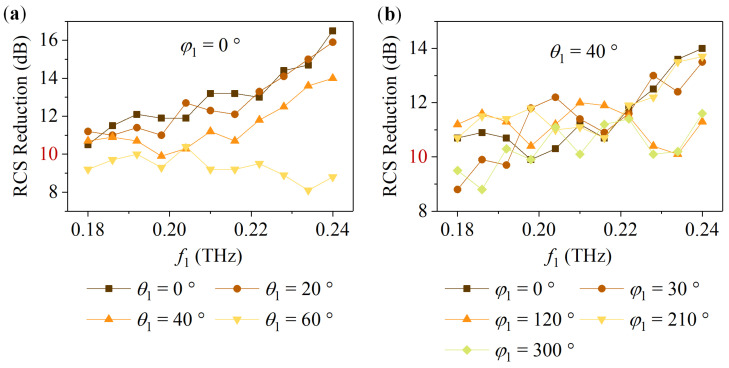
For the metasurface in State 1, the RCS reduction under the LCP oblique incident wave: (**a**) *φ*_1_ = 0° and *θ*_1_ = 0°, 20°, 40°, and 60°; (**b**) *θ*_1_ = 40° and *φ*_1_ = 0°, 30°, 120°, 210°, and 300°.

**Figure 13:**
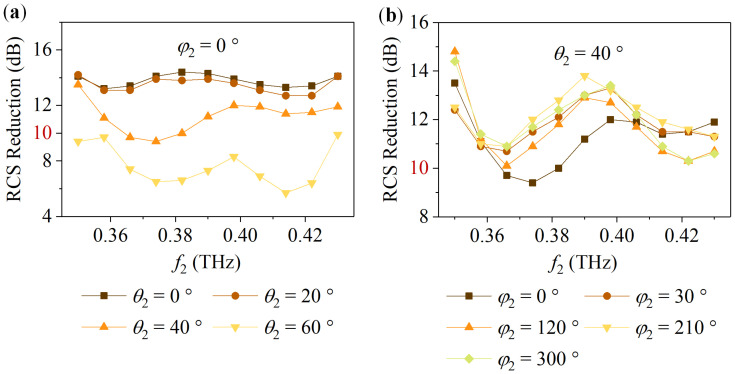
For the metasurface in State 2, the RCS reduction under the RCP oblique incident wave: (**a**) *φ*_2_ = 0° and *θ*_2_ = 0°, 20°, 40°, and 60°; (**b**) *θ*_2_ = 40° and *φ*_2_ = 0°, 30°, 120°, 210°, and 300°.

Further, the broadband and wide-angle characteristics of the metasurface were investigated. For the metasurface in State 1, taking the LCP incident wave as an example, *f*_1_ is in the range of 0.18 THz to 0.24 THz. *θ*_1_ and *φ*_1_ vary from 0° to 60° and 0° to 360°, respectively. Figure 12 shows the simulation result. Figure 12a suggests that for the LCP oblique incident wave with *φ*_1_ = 0° and *θ*_1_ = 0°, 20°, and 40°, the RCS reduction is above 10 dB from 0.18 THz to 0.24 THz. Figure 12b indicates that when the LCP wave with *θ*_1_ = 40° and *φ*_1_ = 0°, 30°, 120°, 210°, and 300° obliquely incidents on the metasurface, the RCS reduction is also generally not less than 10 dB at 0.18 THz to 0.24 THz. For the metasurface in State 2, taking the RCP incident wave as an example, Figure 13 reveals that when *φ*_2_ = 0° with *θ*_2_ changing from 0° to 40° and *θ*_2_ = 40° with *φ*_2_ varying from 0° to 360°, the metasurface can maintain a 10 dB RCS reduction from 0.35 THz to 0.43 THz.

Based on the above results, for the LCP and RCP incident waves with the polar angle and the azimuth angle ranging from 0° to 40° and 0° to 360°, respectively, the effective RCS reduction can be realized at 0.18 THz to 0.24 THz and 0.21 THz to 0.39 THz by tuning VO_2_. Table 1 shows a comparison of this work with previous works. Comparison items include dynamicity, polarization, 10 dB RCS reduction bandwidth (BW), fractional bandwidth (FBW), and max incident angle *θ_i_*. The existing research concentrates on the RCS reduction metasurface with fixed performance working in the microwave band. The proposed VO_2_-based dynamic coding metasurface can realize the terahertz dual-polarized, dual-band, and wide-angle RCS reduction.

## 4. Conclusions

This paper exhibits a method for the terahertz VO_2_-based dynamic coding metasurface. This metasurface can realize a dual-polarized, dual-band, and wide-angle RCS reduction. The meta-atom consists of the resonator, dielectric layer, and metal plate. Two SRRs embedded with two patches of VO_2_ compose the resonator. The simulation results prove that adjusting the state of VO_2_ can effectively change the working frequency band and PB phase of the meta-atom. In addition, limiting the rotation angle to 0° and 90° can introduce the same PB phase of 0° and 180° under the LCP and RCP waves. A 1-bit coding metasurface optimized by the WOA for verification was designed. The simulation results demonstrate that for LCP and RCP vertical incident waves, tuning VO_2_ patches between insulating and metallic states can simultaneously realize an RCS reduction of no less than 10 dB at 0.21 THz and 0.39 THz. Furthermore, the simulation results indicate that for the LCP and RCP oblique incident waves with the polar angle, and the azimuth angle ranging from 0° to 40° and 0° to 360°, respectively, the metasurface can maintain the effective RCS reduction at 0.18 THz to 0.24 THz and 0.21 THz to 0.39 THz. This work has potential value in the terahertz stealth field. Moreover, Surface Micro-Electro-Mechanical Systems (MEMS) technology, micro-nano machining technology, and lithography technology could manufacture the proposed metasurface. Testing the far-field pattern in the THz chamber could verify the performance. Heating could induce the VO_2_ state transition.

## Figures and Tables

**Table 1:** Comparison of this work with previous works.

Ref.	Dynamicity	Polarization	10-dB RCS BW	FBW (%)	Max. *θ_i_* (°)
[12]	No	RCP	10–24 GHz	82.3	60
[3]	No	*x*-pol or *y*-pol	23.71–33.52 GHz (*x*-pol) 25–37.8 GHz (*y*-pol)	34.3 (*x*-pol) 40.8 (*y*-pol)	37
[13]	No	*x*-pol and *y*-pol	7.6–26.2 GHz	110.1	0
[14]	No	*x*-pol and *y*-pol	5–40 GHz	155.6	45
[15]	No	*x*-pol and *y*-pol	3.5–11.5 GHz	106.7	30
[27]	Yes	*x*-pol and *y*-pol	8.52–16.98 GHz	66.4	55
[28]	Yes	*x*-pol and *y*-pol	4.5–13.74 GHz	101.3	30
[29]	Yes	*x*-pol and *y*-pol	3.8–10.73 GHz	95.4	30
This Work	Yes	LCP and RCP	0.18–0.24 THz 0.21–0.39 THz	28.6 60	40

## Data Availability

Data will be made available on request.
